# Quantitative theory of deep brain stimulation of the subthalamic nucleus for the suppression of pathological rhythms in Parkinson’s disease

**DOI:** 10.1371/journal.pcbi.1006217

**Published:** 2018-05-29

**Authors:** Eli J. Müller, Peter A. Robinson

**Affiliations:** 1 School of Physics, The University of Sydney, Sydney, New South Wales, Australia; 2 Center for Integrative Brain Function, The University of Sydney, Sydney, New South Wales, Australia; Oxford University, UNITED KINGDOM

## Abstract

Deep brain stimulation (DBS) of the subthalamic nucleus (STN) is modeled to explore the mechanisms of this effective, but poorly understood, treatment for motor symptoms of drug-refractory Parkinson’s disease and dystonia. First, a neural field model of the corticothalamic-basal ganglia (CTBG) system is developed that reproduces key clinical features of Parkinson’s disease, including its characteristic 4–8 Hz and 13–30 Hz electrophysiological signatures. Deep brain stimulation of the STN is then modeled and shown to suppress the pathological 13–30 Hz (beta) activity for physiologically realistic and optimized stimulus parameters. This supports the idea that suppression of abnormally coherent activity in the CTBG system is a major factor in DBS therapy for Parkinson’s disease, by permitting normal dynamics to resume. At high stimulus intensities, nonlinear effects in the target population mediate wave-wave interactions between resonant beta activity and the stimulus pulse train, leading to complex spectral structure that shows remarkable similarity to that seen in steady-state evoked potential experiments.

## Author summary

Pathological 13-30 Hz (beta) oscillations within the basal ganglia are a characteristic feature of human Parkinson’s disease which seem to correlate with symptom severity. The origin of these oscillations and the suppressive mechanism of effective deep brain stimulation treatments remains to be shown. We formulate a physiologically based population model of the corticothalamic-basal ganglia system that produces 13-30 Hz oscillations in the neural circuit formed between the globus pallidus pars externa and the subthalamic nucleus and the hyperdirect corticothalamic-basal ganglia pathway. We then develop a model of deep brain stimulation applied to the corticothalamic-basal ganglia system that permits systematic determination of effective stimulus protocols, which have been estimated by trial and error to date. Our results demonstrate that high pulse frequency (>140 Hz) stimulation is required to effectively suppress the pathological oscillations, which agrees with clinically used values. Interactions between these oscillations and the applied stimulus also lead to complex spectral structure that shows remarkable similarity to that seen in steady-state evoked potential experiments.

## Introduction

Deep brain stimulation (DBS) has become an effective treatment for a number of neurological disorders such as Parkinson’s disease (PD) and essential tremor [1, 2]. In Parkinson’s disease DBS treatments, a macroelectrode is chronically implanted in a target nucleus, typically either the globus pallidus internus (GPi), subthalamic nucleus (STN), or the ventral intermediate nucleus of the thalamus; this electrode delivers high frequency (>100 Hz) electrical stimulation as a series of pulses. More broadly, studies have also shown the efficacy of DBS treatments in dystonia [3], epilepsy [4], and obsessive-compulsive disorder [5].

Significant progress has been made exploring the influence of DBS on neural activity [6]. However, the efficacy of DBS treatments could be improved with a greater understanding of the underlying therapeutic mechanisms. Furthermore, it is unclear what stimulation parameters, electrode geometries, and electrode locations are most effective for the present and future uses of DBS technologies.

Electrical stimulation of the brain influences a variety of mechanisms involved in the function and signaling of neurons. The sensitivity of different contributing elements depends upon the amplitude and temporal properties of the stimulation [7], geometry of the stimulus field [8], target cell physiology and geometry [9], and the possible pathophysiology of different disease states [10]. It is known that distinct neuron types possess different types of ion channels and that these may have different voltage-sensitive activation and inactivation properties [11]. Thus, the effect of DBS on a single neuron’s dynamics may vary significantly between brain regions. However, by averaging over millimeter to whole-brain scales, a generalized description of DBS at a population level may provide insights into the net effect of these different mechanisms and allow prediction of effective stimulation protocols.

It was initially thought that deep brain stimulation had a predominantly inhibitory effect on the stimulated population due to similar therapeutic effects to lesioning [12, 13]. This inhibition hypothesis is supported by several experimental findings in STN-DBS of rats [14] and monkeys [15] and GPi-DBS and STN-DBS in humans [16, 17, 18]. Furthermore, [19] demonstrated an inhibitory response to GPi-DBS that was mediated by the GABA receptors and that this inhibition could be blocked via a GABA antagonist.

Seemingly in contradiction with the inhibition hypothesis, several experiments with recordings in efferent nuclei of the DBS target population indicate an increase in the stimulated populations activity [20, 21, 22], and an entrainment of local neural firing during DBS [23].

Several modeling approaches have been used to elucidate DBS effects across multiple scales. Finite element methods used in conjunction with multi-compartment neuron models have explored DBS responses in small neural assemblies [24, 25, 8], and the distribution of the applied electric field [26]. These single cell models have demonstrated a disassociation of activity at the soma relative to the axon during extracellular stimulation [24], and a systemic activation of axons both efferent and afferent to the stimulation site [27]. The variable nature of response to DBS between brain regions might then be understood by approximating DBS as an activation of intrinsic axons within a certain effective range of the electrode, and thus could explain observations supporting the contradictory excitation and inhibition hypotheses.

Parkinson’s disease (PD) is a neurodegenerative disorder characterized by motor dysfunction including akinesia, bradykinesia, tremor, and rigidity [28]. These clinical manifestations have been linked to dopaminergic denervation in the substantia nigra pars compacta (SNc) and synucleinopathy leading to Lewy bodies, and neurites in the SNc and other brain regions [29]. The firing pattern hypothesis regarding PD proposes that pathological oscillations and/or synchronization play a primary role in motor symptoms of the disorder. Single unit and local field potential recordings have shown enhanced activity within and between the basal ganglia (BG), thalamus and motor cortex at about 4–8 Hz and 13–30 Hz [30, 31, 32, 33, 34], which seems to correlate with significant coherence at these frequencies [35, 36, 37, 38, 39, 40]. It has thus been suggested that these pathological rhythms cause a disturbance of motor-related information processing in the BG [41], which could explain some PD symptoms.

The enhanced beta band oscillations (13-30 Hz) found in the STN of PD patients are thought to be related to symptom severity, based on direct correlation results [42], as well as observations of a reduction in beta power following treatments that ameliorate PD symptoms, such as dopaminergic supplementation [43] and deep brain stimulation [44]. Several experimental and modelling studies have suggested that the circuit formed between the STN and GPe may be responsible for beta activity generation [45, 46], and that cortical excitatory inputs to the STN amplify them [47]. However, the origin of parkinsonian beta activity is still a matter of debate.

Physiologically based mean-field models of the brain provide a tractable framework for the analysis of large-scale neuronal dynamics by averaging microscopic structure and activity [48, 49, 50, 51, 52]. Neural field theory incorporates realistic anatomy of neural populations, nonlinear neural response, interpopulation connections and dendritic, synaptic, cell-body, and axonal dynamics [48, 50, 52, 53, 54, 55, 56, 57, 58]. Neural field models have been successful in accounting for many characteristic states of brain activity including sleep stages, eyes-open, and eyes-closed in waking, nonlinear seizure dynamics, anesthesia and many other phenomena [52, 53, 55, 51, 59, 60, 61, 62].

In the particular case of Parkinson’s disease, neural field models of the corticothalamic-basal ganglia system have been able to account for several electrophysiological correlates of the disease including changes in population average activity, and ∼4-8 Hz and ∼13–30 Hz oscillations characteristic of EEG and LFP spectra [63, 64, 65]. However, the generative mechanisms of these characteristic PD rhythms is still a matter of debate.

The core aims of this work are to develop a population level description of DBS of the corticothalamic-basal ganglia (CTBG) system that can account for experimental observations and the results of other modeling studies. The work will explore parkinsonian states of the CTBG system and determine whether subthalamo-pallidal circuits can sustain characteristic beta oscillations in this framework. Finally, the effects of DBS on these parkinsonian states will be analyzed and provide insights into the efficacy of DBS treatments.

## Materials and methods

### CTBG model

**Fig 1:**
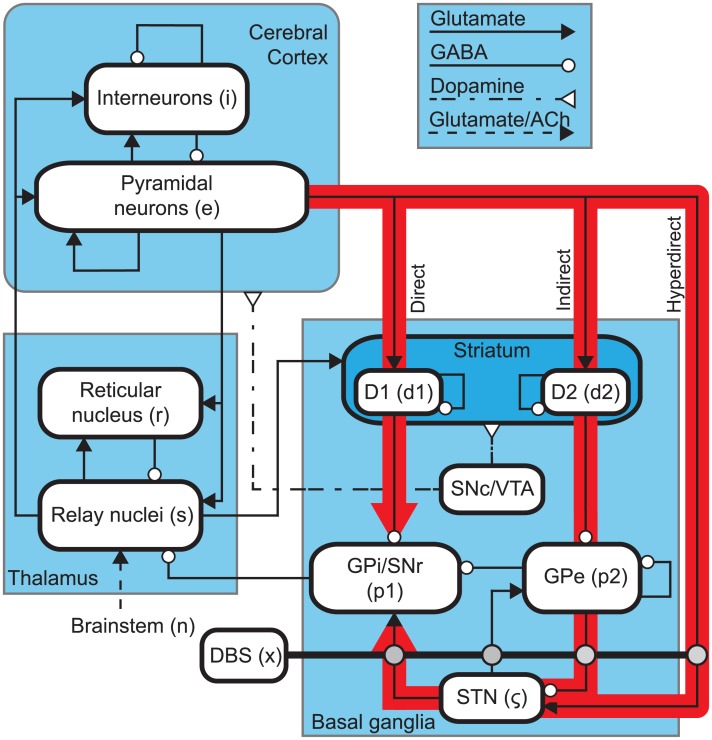
Schematics of the corticothalamic-basal ganglia system. Subscripts used to denote various neural populations are parenthesized. Arrowed lines denote neural connections and corresponding neurotransmitters, glutamate, GABA, dopamine, and acetylcholine (ACh). The population abbreviations are discussed in the Materials and Methods section. Red arrows highlight three key pathways through the basal ganglia.

Fig 1 shows a schematic of the CTBG model. The system contains nine distinct neural populations across three brain regions. The cerebral cortex contains populations of excitatory pyramidal neurons, *e*, and inhibitory interneurons, *i*. The thalamus is divided into an excitatory population for the specific relay nuclei (SRN), *s*, and an inhibitory population for the thalamic reticular nucleus (TRN), *r*. The basal ganglia (BG) contains two inhibitory populations within the striatum, one expressing the D1 dopamine receptor, *d*_1_, and one expressing the D2 dopamine receptor, *d*_2_. The striatum projects to two inhibitory populations, the globus pallidus pars externa, *p*_2_, and a population representing the globus pallidus pars interna and substantia nigra pars reticulata, *p*_1_. The subthalamic nucleus (STN) is represented by an excitatory population, *ζ*. Finally, deep brain stimulation is defined as an input source, *x*, which is coupled to STN as well as to its projection sites. This is discussed in detail in a later section. The substantia nigra pars compacta (SNc) and ventral tegmental area (VTA) are not explicitly defined as a population within the model, however, they are included in Fig 1 as an indication of the neural pathways affected by dopamine.

### Firing rates

The mean firing rate, *Q_a_*(**r**, *t*), of a neural population can be approximately related to its mean membrane potential, *V_a_*(**r**, *t*), by [66, 67]

$$Q_{a}\left(r,t\right)=S_{a}\left[V_{a}\left(r,t\right)\right],$$ (1)

$$=\frac{Q_{a}^{\text{max}}}{1+\text{exp}[-\left\{V_{a}\left(r,t\right)-\theta_{a}\right\}/\sigma^{\prime }]}.$$ (2)

where Eqs (1) & (2) define the sigmoidal mapping function *S*_*a*_, $Q_{a}^{\text{max}}$ is the maximal firing rate, *V*_*a*_ is the average membrane potential relative to resting, *θ*_*a*_ is the mean neural firing threshold, and $\sigma^{\prime }\pi /\sqrt{3}$ is the standard deviation of this threshold.

### Axonal propagation

A number of experimental studies have revealed waves of neural activity spreading across the cortex [68, 49, 69, 70], which have been analyzed theoretically [71, 72, 73, 51, 74, 75, 52, 58]. This propagating activity is represented as a field of mean spike rates in axons, *ϕ*_*a*_. A population *a*, with a mean firing rate *Q*_*a*_, is related to *ϕ*_*a*_ by the damped wave equation

$$\begin{array}{c} D_{a}\left(r,t\right)\phi_{a}\left(r,t\right)=Q_{a}\left(r,t\right), \end{array}$$ (3)

where

$$\begin{array}{c} D_{a}\left(r,t\right)=\frac{1}{\gamma_{a}^{2}}\frac{\partial^{2}}{\partial t^{2}}+\frac{2}{\gamma_{a}}\frac{\partial }{\partial t}+1-r_{a}^{2}\nabla^{2}. \end{array}$$ (4)

Here, *γ*_*a*_ = *v*_*a*_/*r*_*a*_ represents the damping rate, where *v*_*a*_ is the propagation velocity in axons, and *r*_*a*_ is the characteristic axonal length for the population. The propagation of these waves is facilitated primarily by the relatively long-range white matter axons of excitatory cortical pyramidal neurons. Later in our model the simplifying local interaction approximation *r*_*b*_ ≈ 0 is made for *b* = *i*, *r*, *s*, *d*_1_, *d*_2_, *p*_1_, *p*_2_, *ζ* due to the short ranges of the corresponding axons which implies *ϕ_b_*(**r**, *t*) = *Q_b_*(**r**, *t*) for these populations [52, 57, 54, 55, 76, 77].

### Synaptodendritic and somatic response

The mean soma potential *V*_*a*_ of a population *a* at position **r** and time *t* is given by sum of the postsynaptic potentials (PSPs):

$$\begin{array}{c} V_{a}\left(r,t\right)=\sum_{b}V_{ab}\left(r,t\right), \end{array}$$ (5)

where *V*_*ab*_(**r**, *t*) is the postsynaptic potential generated by projections arriving at population *a* from population *b*. The influence of incoming spikes to population *a* from population *b* is weighted by a connection strength parameter, *ν*_*ab*_ = *N*_*ab*_*s*_*ab*_, where *N*_*ab*_ is the mean number of connections between the two populations and *s*_*ab*_ is the mean strength of response in neuron *a* to a single spike from neuron *b*. The postsynaptic potential response in the dendrite is given by

$$\begin{array}{c} D_{\alpha \beta }V_{ab}\left(r,t\right)=\nu_{ab}\left(r,t\right)\phi_{ab}\left(r,t-\tau_{ab}\right), \end{array}$$ (6)

where *τ*_*ab*_ is the average axonal delay for the transmission of signals to population *a* from population *b*. The operator *D*_*αβ*_ describes the time evolution of *V*_*ab*_ in response to synaptic input, and is given by

$$\begin{array}{c} D_{\alpha \beta }=\frac{1}{\alpha \beta }\frac{d^{2}}{dt^{2}}+(\frac{1}{\alpha }+\frac{1}{\beta })\frac{d}{dt}+1. \end{array}$$ (7)

where *β* and *α* are the overall rise and decay response rates to the synaptodendritic and soma dynamics.

### Steady states

It has been shown that nominal brain activity is well characterized by perturbations about a mean value [55]. Hence, we first find the time independent states of the CTBG system. Following the approach of previous neural field models, excitatory and inhibitory synapses in the cortex are assumed proportional to the numbers of neurons [50, 78]. This random connectivity approximation results in *ν*_*ee*_ = *ν*_*ie*_, *ν*_*ei*_ = *ν*_*ii*_, and *ν*_*es*_ = *ν*_*is*_, which implies *V*_*e*_ = *V*_*i*_ and *Q*_*e*_ = *Q*_*i*_. Inhibitory population variables can then be expressed in terms of excitatory quantities and are thus not neglected even though they do not appear explicitly below.

The steady states are obtained by setting all time derivatives to zero in Eqs (3), (4) and (6). Using the connectivity shown in Fig 1, and excluding DBS, Eqs (5) and (6) give

$$\begin{array}{cc} & V_{e}^{\left(0\right)}=\left(\nu_{ee}+\nu_{ei}\right)\phi_{e}^{\left(0\right)}+\nu_{es}\phi_{s}^{\left(0\right)}, \end{array}$$ (8)

$$\begin{array}{cc} & V_{r}^{\left(0\right)}=\nu_{re}\phi_{e}^{\left(0\right)}+\nu_{rs}\phi_{s}^{\left(0\right)}, \end{array}$$ (9)

$$\begin{array}{cc} & V_{s}^{\left(0\right)}=\nu_{se}\phi_{e}^{\left(0\right)}+\nu_{sr}\phi_{r}^{\left(0\right)}+\nu_{sp_{1}}\phi_{p_{1}}^{\left(0\right)}+\nu_{sn}\phi_{n}^{\left(0\right)}, \end{array}$$ (10)

$$\begin{array}{cc} & V_{d_{1}}^{\left(0\right)}=\nu_{d_{1}e}\phi_{e}^{\left(0\right)}+\nu_{d_{1}s}\phi_{s}^{\left(0\right)}+\nu_{d_{1}d_{1}}\phi_{d_{1}}^{\left(0\right)}, \end{array}$$ (11)

$$\begin{array}{cc} & V_{d_{2}}^{\left(0\right)}=\nu_{d_{2}e}\phi_{e}^{\left(0\right)}+\nu_{d_{2}s}\phi_{s}^{\left(0\right)}+\nu_{d_{2}d_{2}}\phi_{d_{2}}^{\left(0\right)}, \end{array}$$ (12)

$$\begin{array}{cc} & V_{p_{1}}^{\left(0\right)}=\nu_{p_{1}\zeta }\phi_{\zeta }^{\left(0\right)}+\nu_{p_{1}d_{1}}\phi_{d_{1}}^{\left(0\right)}+\nu_{p_{1}p_{2}}\phi_{p_{2}}^{\left(0\right)}, \end{array}$$ (13)

$$\begin{array}{cc} & V_{p_{2}}^{\left(0\right)}=\nu_{p_{2}\zeta }\phi_{\zeta }^{\left(0\right)}+\nu_{p_{2}d_{2}}\phi_{d_{2}}^{\left(0\right)}+\nu_{p_{2}p_{2}}\phi_{p_{2}}^{\left(0\right)}, \end{array}$$ (14)

$$\begin{array}{cc} & V_{\zeta }^{\left(0\right)}=\nu_{\zeta e}\phi_{e}^{\left(0\right)}+\nu_{\zeta p_{2}}\phi_{p_{2}}^{\left(0\right)}. \end{array}$$ (15)

The system’s steady states then can be determined by considering the simultaneous zeros of the five functions

$$\begin{array}{cc} & F\left(\phi_{e}\right)=\phi_{e}-S_{e}[\left(\nu_{ee}+\nu_{ei}\right)\phi_{e}+\nu_{es}\phi_{s}], \end{array}$$ (16)

$$\begin{array}{cc} & F\left(\phi_{s}\right)=\phi_{s}-S_{s}[\nu_{se}\phi_{e}+\nu_{sr}\phi_{r}+\nu_{sp_{1}}\phi_{p_{1}}+\nu_{sn}\phi_{n}], \end{array}$$ (17)

$$\begin{array}{cc} & F\left(\phi_{d_{1}}\right)=\phi_{d_{1}}-S_{d_{1}}[\nu_{d_{1}e}\phi_{e}+\nu_{d_{1}s}\phi_{s}+\nu_{d_{1}d_{1}}\phi_{d_{1}}], \end{array}$$ (18)

$$\begin{array}{cc} & F\left(\phi_{d_{2}}\right)=\phi_{d_{2}}-S_{d_{2}}[\nu_{d_{2}e}\phi_{e}+\nu_{d_{2}s}\phi_{s}+\nu_{d_{2}d_{2}}\phi_{d_{2}}], \end{array}$$ (19)

$$\begin{array}{cc} & F\left(\phi_{p_{2}}\right)=\phi_{p_{2}}-S_{p_{2}}[\nu_{p_{2}d_{2}}\phi_{d_{2}}+\nu_{p_{2}p_{2}}\phi_{p_{2}}+\nu_{p_{2}\zeta }\phi_{\zeta }]. \end{array}$$ (20)

where *ϕ*_*r*_, *ϕ_p1_*, and *ϕ*_*ζ*_ can be determined from Eqs (9), (13) and (15), respectively, in conjunction with Eq (1). The roots of Eqs (16)–(20) are computed numerically using the MATLAB function fsolve() with a tolerance of 10^−15^ V.

### Resonances and gains

A linearized form of the CTBG model can be used to derive the transfer function of the system [63, 64, 65]. This is a function of the internal gains of the system, which represent the additional activity generated in postsynaptic nuclei per additional unit input activity from presynaptic nuclei, and are [53, 55]

$$\begin{array}{c} G_{ab}=\rho_{a}\nu_{ab} \end{array}$$ (21)

where

$$\rho_{a}={\frac{dQ_{a}}{dV_{a}}|}_{V_{a}^{\left(0\right)}}=\frac{\phi_{a}^{\left(0\right)}}{\sigma^{\prime }}\left[1-\frac{\phi_{a}^{\left(0\right)}}{Q_{a}^{\text{max}}}\right].$$ (22)

### Numerical simulations

All numerical simulations of the CTBG neural field model in this work are performed using the NFTsim code package detailed by [79]. This package is used to solve Eqs (1)–(7) numerically for the spatially uniform case where the ∇^2^ in (4) is zero. The solutions to these delay differential equations are found using a standard forth-order Runge-Kutta integration method with a time step of 10^−4^ s.

Nominal brain states have been found to exist near stable fixed points [55]. Hence, all simulations in this work are performed with the system initialized to the low firing steady state found in the previous section using the parameters given in Table 1, unless otherwise specified.

**Table 1:** Nominal parkinsonian parameters adapted from [63].

Quantity	Value	Unit
*r*	80	mm
*σ*′	3.3	mV
$\phi_{n}^{\left(0\right)}$	1	s^−1^
*γ*_*e*_	116	s^−1^
*α*	50	s^−1^
*β*	200	s^−1^
*τ*_*re*_, *τ*_*se*_	45	ms
*τ*_*es*_	35	ms
$Q_{e}^{\text{max}}$, $Q_{r}^{\text{max}}$, $Q_{p_{2}}^{\text{max}}$	300	s^−1^
$Q_{d_{1}}^{\text{max}}$, $Q_{d_{2}}^{\text{max}}$	65	s^−1^
$Q_{p_{1}}^{\text{max}}$	250	s^−1^
$Q_{\zeta }^{\text{max}}$	500	s^−1^
*θ*_*e*_	14	mV
*θ*_*r*_, *θ*_*s*_	13	mV
$\theta_{d_{1}}$, $\theta_{d_{2}}$	19	mV
$\theta_{p_{1}}$, *θ*_*ζ*_	10	mV
$\theta_{p_{2}}$	9	mV
*ν*_*ee*_	1.2	mV s
*ν*_*ei*_	−1.5	mV s
*ν*_*es*_	1.1	mV s
*ν*_*re*_	0.1	mV s
*ν*_*rs*_	0.1	mV s
*ν*_*se*_	1.5	mV s
*ν*_*sr*_	−0.1	mV s
$\nu_{sp_{1}}$	−0.2	mV s
*ν*_*sn*_	0.5	mV s
$v_{d_{1}}{}_{e}$	0.1	mV s
$v_{d_{1}}{}_{s}$	1	mV s
$\nu_{d_{1}d_{1}}$	−0.02	mV s
$v_{d_{2}}{}_{e}$	0.1	mV s
$v_{d_{2}}{}_{s}$	0.1	mV s
$\nu_{d_{2}d_{2}}$	−0.02	mV s
$\nu_{p_{1}d_{1}}$	−0.2	mV s
$\nu_{p_{1}p_{2}}$	−0.02	mV s
$v_{p_{1}}{}_{\zeta }$	1	mV s
$\nu_{p_{2}d_{2}}$	−0.8	mV s
$\nu_{p_{2}p_{2}}$	−0.2	mV s
$v_{p_{2}}{}_{\zeta }$	2.4	mV s
*ν*_*ζe*_	1.3	mV s
$\nu_{\zeta p_{2}}$	−0.2	mV s

### Modeling DBS

Many different stimulus protocols have been used in clinical DBS—with different pulse geometries (i.e. sinusiodal or square-wave), signal amplitudes, stimulation frequencies, and/or transient stimulation phases, followed by varied quiescent periods.

In this work we seek a general formulation of a neural populations response to fluctuations in an applied electric field that will allow for the effects of various stimulus protocols to be determined.

The minimum current necessary to stimulate a given neural element with a long stimulus duration is called the rheobase [80]. The minimum length of time required to activate a given neural element using a stimulus amplitude twice as large as the rheobase is called the chronaxie. Extracellular stimulation experiments have demonstrated a chronaxie time for the myelinated axons which is substantially smaller than the chronaxies of the cell body and dendrites [7, 81, 82]. Hence, our key assumption is that the net effect of fluctuations of an applied electric field is a stimulation of voltage-gated ion channels that induces transmembrane current flow predominantly in both afferent and efferent axons of a subset of neurons within the stimulated population.

A mean-field model has recently been used to describe population effects of transcranial magnetic stimulation [83, 84]. A modification of this approach is used by defining an external pulse rate *ϕ*_*x*_(*t*) that consists of a train of pulses with a width *t*_width_ similar to time series used in DBS treatments. The applied stimulation is then given by

$$\begin{array}{c} \phi_{x}\left(t\right)=\phi_{x}^{\text{max}}\sum_{j}R\left(t-t_{j}^{p}\right), \end{array}$$ (23)

where $\phi_{x}^{\text{max}}$ is the pulse amplitude and *R*(*t*) is a top-hat function of width *t*_width_,

$$\begin{array}{c} R\left(t\right)=\{\begin{array}{cc} 1, & 0<t<t_{\text{width}},\\ 0, & \text{otherwise.} \end{array} \end{array}$$ (24)

The time-integral of *ϕ*_*x*_(*t*), Eq (23), over the pulse width *t*_width_ is the average number of additional spikes generated in the target axon by the applied stimulation. The external stimulus is then coupled to a target population *a* via a connection parameter *ν*_*ax*_ with a pulse frequency *f*_stim_. In the case of STN-DBS, *ϕ*_*x*_(*t*) is coupled to the STN, but also to the GPi and GPe populations as an approximation of the activation of axons terminals near the stimulation site.

## Results

### Effects of stimulation in the model

An afferent spike rate to any population in the CTBG system induces a change in the dendritic membrane potential of that population with a time evolution described by Eq (6). Depending on the connection type, this change may be positive (excitatory) or negative (inhibitory). Each inter-population connection then produces a change in voltage which is integrated at the soma, as described by Eq (5).

**Fig 2:**
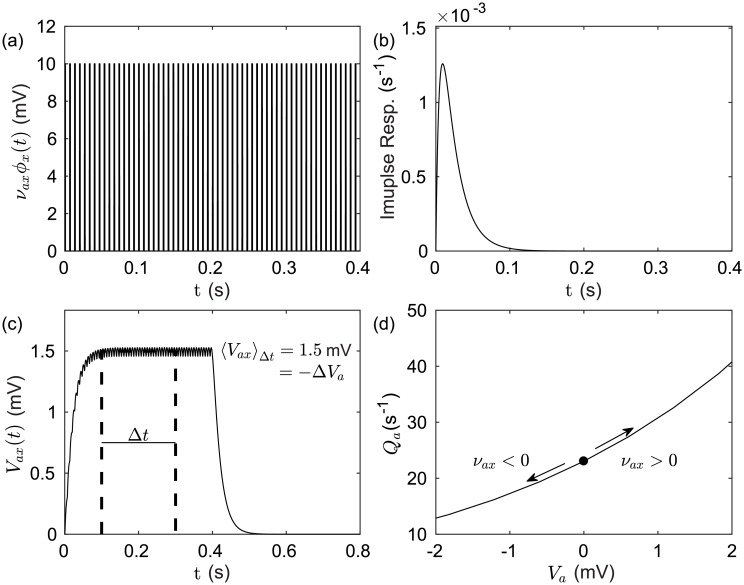
Effects of external pulse train stimulus on population activity. (a) An example evoked response time series for an external pulse frequency *f*_stim_ = 130 Hz, pulse width *t*_width_ = 130 ms, and coupling strength *ν*_*ax*_ = 10 mV s. (b) Impulse response for a unit input at *t* = 0 with decay and rise rate parameters, *β* = 200 s^−1^ and *α* = 50 s^−1^, respectively. (c) Evoked response potential of a given population to the external stimulus represented by the convolution of the impulse response with the stimulus time series. (d) The mean effect of a voltage perturbation evoked by an external stimulus on the distribution of firing rates with a population. Example parameters used are *Q*^max^ = 500 s^−1^, *σ*′ = 3.3 mm, and *θ*_*a*_ = 10 mV.

In the case of DBS, *ϕ*_*x*_(*t*), the mean voltage perturbation observed at the soma of neurons, can be shown by numerically convolving the stimulus time series with the normalized impulse response function given in differential form in Eq (7). Fig 2 shows the evoked response potential generated by a stimulus pulse train, which resembles typical 130 Hz clinical stimulation, and the resulting perturbation to the target population firing rate. The temporal parameters used for the stimulus in Fig 2(a) prescribes an inter-pulse quiescent period of about 7 ms. It can be seen in Fig 2(b) that during this period the impulse response function only decays to about 80% of its maximum value. Fig 2(c) can then be understood as showing small oscillations in the evoked response potential about a constant mean perturbation that results from stimulus time scales which are shorter than population response time scales. The evoked response potential is integrated at the soma of the target population along with intrinsic afferents from other populations within the network. A constant perturbation applied to the soma potential of a population changes its mean firing rate by moving the population along its corresponding sigmoidal response function, (1). Fig 2(d) demonstrates that in the case of inhibition mean soma potentials correspond to lower mean firing rates when compared with unperturbed values. In the case of excitation, the effect is reversed with mean soma potentials corresponding to higher mean firing rates when compared with the unperturbed values.

### Parkinsonian oscillations

Enhanced activity at ∼13-30 Hz is a common feature of Parkinson’s disease patient LFP recordings in the GPi and STN which has been correlated with symptom severity [43, 44, 42]. Recent works have suggested that the neural circuit formed between the GPe and STN can generate these beta oscillations [45, 46] and that excitatory inputs from the cortex may facilitate their amplification [47].

Table 1 contains parameter estimates for parkinsonian states of the CTBG model adapted from [63]. Changes to the [63] connection strength estimates were made in order to explore the effects of a dominant GPe-STN-GPe pathway.

**Fig 3:**
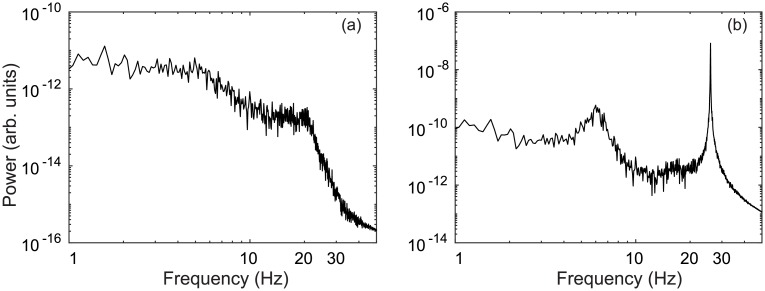
Parkinsonian activity in the STN. (a) Power spectrum of STN firing rate *ϕ*_*ζ*_ for $v_{p_{2}}{}_{\zeta }=1.8$ mVs. (b) Power spectrum of STN firing rate *ϕ*_*ζ*_ for $v_{p_{2}}{}_{\zeta }=2.4$ mVs.

In Fig 3(a) and 3(b), power spectra of the STN firing rate demonstrate enhanced activity at ∼26 Hz, as well as at ∼6 Hz. By increasing STN-GPe coupling $v_{p_{2}}{}_{\zeta }$, damping of the GPe-STN-GPe loop is weakened and results in a strengthened hyperdirect pathway. Together these loops drive 26 Hz oscillations in the STN firing rate which project to the GPi population through STN efferents and then on to thalamic and cortical populations.

The 6 Hz STN oscillations observed in Fig 3(b) are weaker than the 26 Hz beta oscillations. However, the power spectrum for the cortical population shows an opposite relationship with stronger 6 Hz activity than the 26 Hz beta oscillations. This is an interesting result because tremor oscillations in PD patients measured via electromyography (EMG) are typically about 6 Hz and these correlate well with motor cortical activity measured via electroencephalography (EEG) [85]. However, STN LFP recordings about the 4–6 Hz tremor frequency have yet to be demonstrated as a reliable source for tremor detection [86]. Furthermore, other studies have suggested the thalamo-basal ganglia circuit as the origin of tremor oscillations [87].

The model configuration required to produce a dominant GPe-STN-GPe loop resonance involves both an increase in STN-GPe coupling $v_{p_{2}}{}_{\zeta }$, with respect to previous parameter estimates [63], as well as an increase in cortico-STN coupling *ν*_*ζe*_. This is consistent the findings of a recent conductance-based modelling study where cortical inputs amplified parkinsonian oscillations generated by the subthalamo-pallidal circuit [47], although the frequencies observed in that study were lower, 8–14 Hz, and represent dopamine depleted states of primates [45].

### Suppression of PD oscillations by DBS

In this section the CTBG system is numerically simulated using parkinsonian parameters defined in Table 1. These parameters yield strong GPe-STN and hyperdirect loop resonances, which results in large amplitude ∼26 Hz oscillations in STN activity.

**Fig 4:**
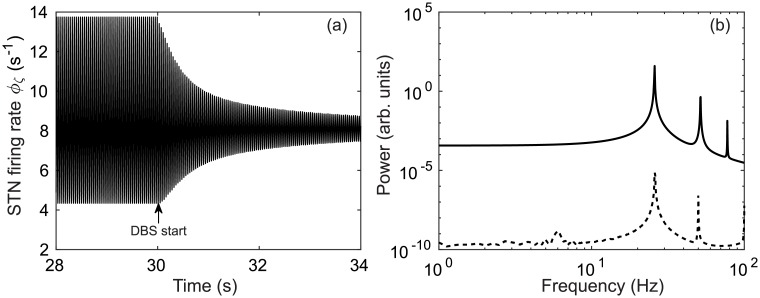
Suppression of parkinsonian beta activity for a 150 Hz external stimulus applied at *t* = 30 s with *ν*_*ζx*_ = −1.2 mVs and $v_{p_{1}x},v_{p_{1}x}=1.2$ mVs. (a) Time series of STN firing rate *ϕ*_*ζ*_ with DBS stimulus applied at *t* = 30 s. (b) Power spectrum of STN firing rate *ϕ*_*ζ*_ before DBS application using the time window *t* = 10–30 s (solid), and during DBS application using a time window *t* = 30–50 s (dashed).

In Fig 4(a) parkinsonian ∼26 Hz STN activity is simulated for 30 s and then 150 Hz DBS is applied. Following the application of this stimulation, a damping of the ∼26 Hz oscillation is observed. A comparison of STN power spectrums pre-stimulus and during stimulation is given in Fig 4(b) and this shows peak power is reduced as a result of DBS.

**Fig 5:**
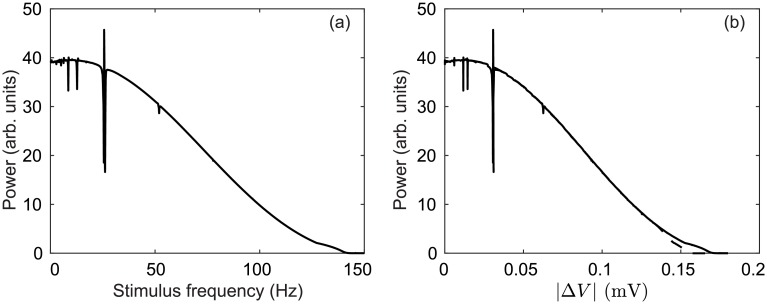
Peak power of STN firing rate *ϕ*_*ζ*_ during a 40 s simulation within the frequency band *f* = 20–30 Hz using a time window *t* = 20–40 s. (a) Dependence of peak *f* = 20–30 Hz power on DBS pulse frequency with *ν*_*ζx*_ = −1.2 mVs and $v_{p_{1}x},v_{p_{2}x}=1.2$ mVs. (b) Change in peak *f* = 20–30 Hz power by a direct constant perturbation to the soma potential (dashed) and by an indirect perturbation resulting from an oscillating DBS input (solid), as described in Fig 2. For each point *V*_*ζ*_ = *V*_*ζ*_ + Δ*V*, $V_{p_{1}}=V_{p_{1}}-\Delta V$, and $V_{p_{2}}=V_{p_{2}}-\Delta V$.

Fig 5(a) demonstrates that increasing DBS pulse frequency strengthens the suppression of 13-30 Hz STN activity. As discussed in previous sections, coupling a DBS input to any population in the model results in an effective constant perturbation to the membrane potential *V*_*a*_ of that population. Because DBS is coupled to the STN, GPe, and GPi populations via *ν*_*ζx*_ = −1.2 mVs and $v_{p_{1}x},v_{p_{1}x}=1.2$ mVs, each corresponding mean membrane potential is perturbed by |Δ*V*|. This perturbation is −Δ*V* (inhibitory) for the STN population, and +Δ*V* (excitatory) for the GPi and GPe populations. In Fig 5(b) we compare power suppression at 13–30 Hz for two cases: In the first case, a direct constant perturbation is made to the membrane potentials of the STN, GPi, and GPe populations. In the second case, the stimulus input used to produce Fig 5(a) is convolved with an impulse response function, as discussed in a previous section. This allows the DBS input to be approximately represented as a constant perturbation to the membrane potential of a given population. Fig 5(b) shows how peak power between 13-30 Hz is effected by directly perturbing the mean membrane potential for the STN, GPi, and GPe populations relative to indirectly perturbing them with an oscillating DBS input. The suppression of pathological beta activity by DBS in our model is then largely attributable to this effective perturbation to the mean membrane potential.

Fig 5(a) and 5(b) also show constructive wave interactions for stimulus pulse frequencies equal to the 26 Hz beta oscillation and destructive interactions near the beta peak and its harmonic (52 Hz) and subharmonic (13 Hz). Studies have shown low-frequency stimulation may worsen PD motor symptoms [88, 89] as well as improve them [90].

**Fig 6:**
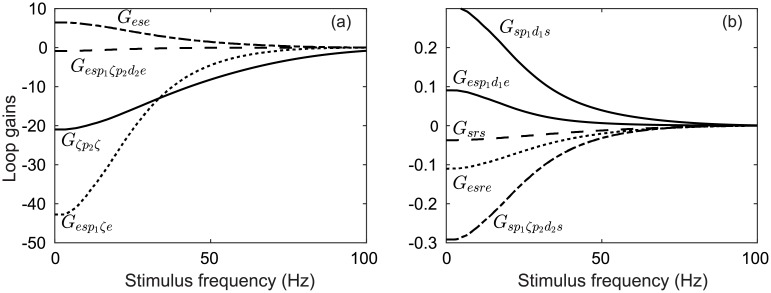
Perturbation of key CTBG loop gains during DBS with *ν*_*ζx*_ = −1.2 mVs, $v_{p_{1}x},v_{p_{1}x}=1.2$ mVs, and all other parameters as defined in Table 1. (a) Dependence of loop gains on the DBS pulse frequency. (b) Dependence of loop gains on the DBS pulse frequency.

The dependence of key network gains on the DBS pulse frequency is shown in Fig 6(a) and 6(b). The parkinsonian parameters define a pathologically strong STN-GPe-STN loop gain as well as a strong hyperdirect pathway. As the pulse frequency of the DBS inputs increases, so too does the net inhibition in the system. This is due to DBS inputs activating the inhibitory pallidal populations (GPe and GPi) more strongly. In contrast, the remaining DBS input inhibits the STN population, which is critical to the generation of a ∼ 26 Hz resonance.

It is important to note that the same suppressive effect can be achieved for a lower stimulus frequency if the stimulus amplitude is correspondingly increased. In DBS treatments, using larger signal amplitudes has the potential to increase the area directly affected by the applied stimulation, possibly incorporating non-motor projecting segments of the STN or even adjacent populations. Our results demonstrate that a high stimulus pulse frequency (*f*_stim_ > 100 Hz) is necessary for beta suppression when the signal amplitude is constrained to be small relative to other STN inputs, e.g., for the Table 1 parameters DBS constitutes ∼6% of the connection weighted activity arriving at the STN over a time interval greater than several stimulus pulse widths.

**Fig 7:**
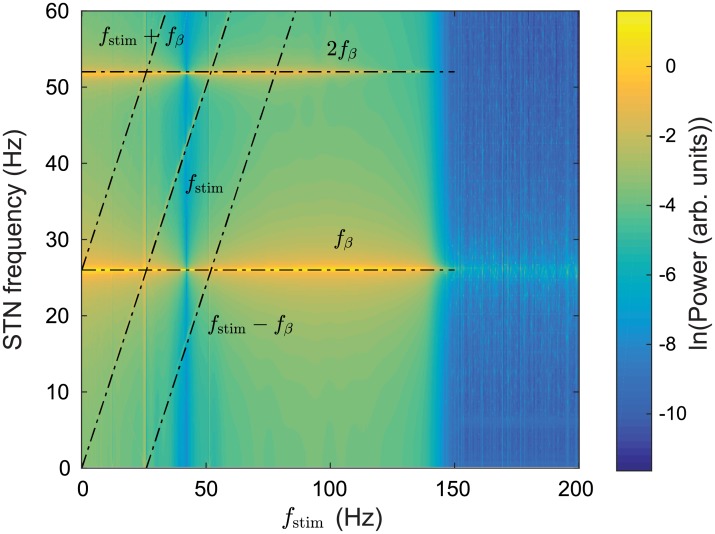
Power spectrum of parkinsonian STN activity as a function of DBS pulse frequency. A simulation of 40 s is used with *ν*_*ζx*_ = −1.2 mVs and $v_{p_{1}x},v_{p_{2}x}=1.2$ mVs and a power spectrum of the STN firing rate is calculated over the time window *t* = 20–40 s.

Fig 7 shows the power spectrum of the STN firing rate time series as a function of DBS pulse frequency. Strong oscillations are seen at ∼26 Hz and its second harmonic ∼52 Hz. When the stimulus pulse frequency reaches about 140 Hz the ∼26 Hz power decreases by several orders of magnitude. Overall power within the 0–60 Hz frequency band also decreases and is redistributed to higher frequencies.

Additionally, Fig 7 shows peaks at the stimulus frequency *f*_stim_ (1:1) and also at its harmonics *f*_stim_(N:1), however, the harmonics are much weaker and not clearly discernible in this plot.

**Fig 8:**
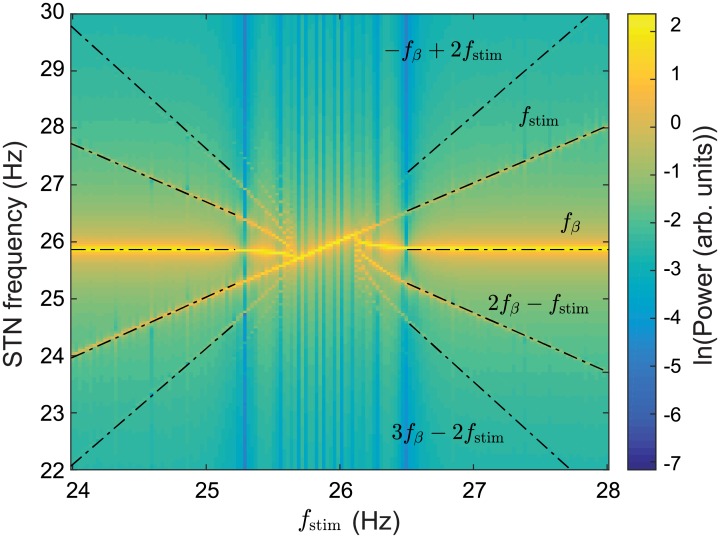
Power spectrum of parkinsonian STN activity as a function of DBS pulse frequency with *f*_stim_ = 24–28 Hz. A simulation of 40 s is used with *ν*_*ζx*_ = −1.2 mVs and $v_{p_{1}x},v_{p_{2}x}=1.2$ mVs and the power spectrum of the STN firing rate is calculated over the time window *t* = 20–40 s.

Nonlinear wave interactions have previously been demonstrated in a neural field model of the corticothalamic system [91] which shows good agreement with human EEG studies [92]. In [91], a periodic nonlinear input was used to drive the CT system and resulted in nonlinear interactions between the drive frequency and an intrinsic alpha oscillation. Spectral peaks were found at frequencies equal to the sum and difference of the drive frequency and the intrinsic alpha frequency, *f*_±_ = |*f*_stim_ ± *f_α_*|, as well their respective harmonics. Our model also demonstrates these nonlinear interactions. In Fig 7, spectral peaks are seen at the sum and difference of the stimulus pulse frequency and the beta frequency *f*_±_ = |*f*_stim_ ± *f_β_*| where *f*_*β*_ = 26 Hz. These nonlinear interactions are much more distinct in Fig 8 with peaks seen at *f*_±_ = −*f*_stim_ + 2*f_β_*, 2*f*_stim_ − *f_β_*, and −2*f*_stim_ + 3*f*_β_. Additionally, Fig 8 demonstrates an entrainment of STN activity as a result of DBS inputs. The intrinsic parkinsonian beta peak is shifted to match the stimulus pulse frequency within the 25.5-26.2 Hz range. This result is consistent with experimental findings in human PD studies where a local entrainment of neural activity was observed during GPi-DBS [23].

## Discussion

In this work we have developed a novel description of deep brain stimulation and incorporated it into a neural field model of the corticothalamic-basal ganglia system. The model has enabled us to explore generative mechanisms for the pathological beta band activity observed in Parkinson’s disease and the influences of DBS on these oscillations. The main results of the paper are as follows:

1. A general description of deep brain stimulation was developed and applied to a neural field model of the CTBG system originally proposed by [63] and which is further developed in this work. The model provides a framework for exploring characteristic states of Parkinson’s disease and the influence of DBS protocols on these states.
2. STN-DBS was found to produce an effective constant perturbation to the membrane potentials of the STN and pallidal populations, which have network-wide effects on stationary states and interpopulation connection gains. The impact these perturbations have on the system gains and population activity is dependent on the prestimulus state of the system. This implies that identical stimulation protocols applied to the same region of the brain in two individuals may have distinct impacts on brain activity. In the case of PD, patient specific variations of the pathophysiology may contribute to diverse stimulus response. Thus, it is essential that prestimulus brain states be better understood in order to improve the efficacy of DBS treatments and future uses.
3. Beta activity around 26 Hz was generated via a pathologically strong subthalamo-pallidal-subthalamic loop which resulted in a dominant indirect pathway through the basal ganglia. The beta oscillations are consistent with coherence peaks between the subthalamic nucleus and globus pallidus observed experimentally [30, 37]. The dependence of these parkinsonian oscillations on the STN-GPe-STN pathway is also consistent with the results of a reduced CTBG model [46] and experiments with monkeys rendered parkinsonian [45].
4. It was found that cortical inputs to the STN population can amplify the pathological beta activity which has been suggested by experiments using antagonists to block cortical projections to the STN [45]. This amplification has also been demonstrated in a conductance-based modeling study using ∼250 STN and GPe neurons [47]. Our model generalizes this result by incorporating realistic inputs from the striatum to the pallidal populations, as well as direct excitatory inputs from the cortex to the STN.
5. 6 Hz oscillations were observed in STN activity which projected more strongly to the cortical populations than the beta activity and are the result of a pathologically large cortico-basal ganglia-thalamo-cortical pathway. The result provides a possible generative mechanism for 6 Hz tremor oscillations in PD patients which correlate well with motor cortical activity [85]. However, STN LFP recordings about the 4–6 Hz tremor frequency have yet to be demonstrated as a reliable source for tremor detection [86] and studies have suggested the thalamo-basal ganglia circuit may be the origin of tremor oscillations [87].
6. High pulse frequency (> 140 Hz) DBS of the STN was effective in suppressing pathological 26 Hz activity characteristic of Parkinson’s disease. The suppressive effect was shown to result from a perturbation to the mean membrane potential of the STN, and the external and internal segments on the globus pallidus. Low frequency stimulation in human PD has so far failed to show optimal symptom control [88, 93]. This could be due to an insufficient amount of DBS evoked activity required for damping the resonance responsible for pathological beta activity. Larger stimulus amplitudes result in a larger volume of directly influenced neurons, potentially extending beyond the motor related regions of the STN, and even adjacent brain regions. Therefore, in order to achieve the necessary levels of evoked activity for damping the system resonance, higher pulse frequencies are required.
7. Nonlinear interactions were found to occur between the parkinsonian beta resonance and stimulus pulse frequency, which resulted in an entrainment of STN activity. This supports experimental findings in human PD studies where a local entrainment of neural activity was observed during GPi-DBS [23].
8. Stimulation frequencies equal to the beta frequency peak resulted in enhanced beta power in STN activity via a constructive wave interaction. Studies have suggested beta band stimulation may worsen PD motor symptoms [88] while 60-80 Hz stimulation may improve them [90].

Overall, our work provides insights into the generative mechanisms of pathological oscillations in human Parkinson’s disease and the population level effects of deep brain stimulation upon these oscillations. Furthermore, the model provides a framework for predicting effective stimulus protocols systematically rather than by trail and error, as has been the case to date.

Closed-loop adaptive DBS systems use feedback from local field potential measurements made via the implanted simulation electrode to modulate stimulus protocols [94]. Our model could be used in conjunction with an adaptive DBS system to increase the efficacy of clinical treatments. Cortical and subthalamic firing rate spectra in this model could be fitted to EEG and LFP spectra during an on-off DBS treatment cycle. The change in spectra corresponds to specific variable changes in the model and the trajectory of these changes could then be used as a detection method for parkinsonian states that are specific to the patient.

Several studies have observed antidromic activation as a result of deep brain stimulation [95], and activation of pallido-thalamic fibers during STN-DBS [96], which could be included in future generations of the model.
